# Social capital: Implications for neurology

**DOI:** 10.1002/brb3.1169

**Published:** 2018-12-08

**Authors:** Saúl Reyes, Gavin Giovannoni, Alison Thomson

**Affiliations:** ^1^ Queen Mary University of London, Blizard Institute London UK; ^2^ Barts and The London School of Medicine and Dentistry London UK

**Keywords:** neurology, public health, social capital, social networks, socioeconomic factors

## Abstract

Social capital (SC) is a broad term that encompasses the many resources derived from social connections. The contemporary study of SC in public health has deep roots in the related fields of sociology, economics, and politics. Its multidisciplinary nature and the varying potential ways it could affect individuals have resulted in different but overlapping models to approach SC in the health field. There are currently no standardized measures of SC, and even more challenging its impact on health outcomes seems to vary according to the level of analysis. Despite the accumulating evidence that supports a protective effect of SC on mental and physical health, and mortality, not enough attention has been paid to the potential drawbacks of SC. The role of SC in neurological disease is just beginning to be explored. Concerted efforts are needed to ensure that empirical evidence on SC could be properly translated into interventions for health‐promoting purposes. In this paper, we review the current state of scientific knowledge on the subject of SC, with a focus on its application in the field of neurology.

## 1 INTRODUCTION

Although the impact of social environment on health outcomes is well documented, there is no consensus as to the relative importance of each factor. Many determinants are involved, and among them, social capital (SC) has emerged as one of the most interesting old ideas being revisited from a new perspective (Pearce & Davey Smith, 2003). SC refers to the many resources derived from the social interaction between individuals and groups. It can be considered as a by‐product of social relationships that facilitates coordination and cooperation for mutual benefit (Bourdieu, 1986; Islam, Merlo, Kawachi, Lindström, & Gerdtham, 2006; Kawachi, Kennedy, Lochner, & Prothrow‐Stith, 1997). Such a broad definition is controversial but necessary to understand how SC is shaped by a wide variety of social aspects, including networks, organizations, norms of reciprocity, and social trust within a community. The distinction between different forms of SC, that is, cognitive, structural, bonding, bridging, and linking, has helped researchers to better understand its multidimensional nature and its effect on individual and public health (Egan, Tannahill, Petticrew, & Thomas, 2008; Ehsan & De Silva, 2015; Gilbert, Quinn, Goodman, Butler, & Wallace, 2013; Nyqvist, Pape, Pellfolk, Forsman, & Wahlbeck, 2014). More recently, the concept of SC has become the subject of intense discussion, as it may represent a pathway by which public health interventions lead to health improvement (Coll‐Planas et al., 2017; Eriksson, 2011; Story, 2013). However, the exact nature and magnitude of these effects remain controversial, as there are no standardized tools to measure SC.

Neurological disorders represent a large burden on worldwide health (Murray et al., 2012). Patients with neurological conditions are embedded in social structures that may affect their outcomes. This effect has been postulated to modify the risk of dementia and the long‐term prognosis in patients with stroke (Dhand, Luke, Lang, & Lee, 2016). Regardless of the growing recognition of the role of SC in chronic diseases, not enough attention has been paid to its potential impact on many disabling neurological conditions. In this review, we describe the current state of the SC model and its implications in the field of neurology.

## 2 WHAT IS SOCIAL CAPITAL?

The historical background and changing definitions of SC over the last few decades are critical in improving our understanding of the complexity of its conceptualization and operationalization.

The SC concept emerged in sociology early in the past century, beginning with Lyda Hanifan's publication on “The community center.” She defined SC in a figurative sense as “the resources in life which tend to make tangible substances count for most in the daily lives of a people; namely, goodwill, fellowship, sympathy, and social intercourse among the individual and families who make up a social unit” (Hanifan, 1920). Since then, other sociologists contributed to the inclusion of the SC perspective in the study of communities and social transformation (Bourdieu, 1980; Coleman, 1988; Jacobs, 1961).

After these foundational principles were grasped, the concept of SC was incorporated in public health. Review and research articles exploring the impact of social relationships on population health are closely linked as intellectual sources for the translation and conceptualization of SC into public health research and practice (Berkman & Syme, 1979; Cohen, 1988; Cohen & Wills, 1985; Moore, Haines, Hawe, & Shiell, 2006). More recently, Putnam expanded the theory of SC to a collective level, which also marked and important moment in public health's application and acceptance of the concept. According to Putnam, SC is defined as “features of social organization, such as trust, norms, and networks, that can improve the efficiency of society by facilitating coordinated actions” (Putnam, 2000; Putnam, Leonardi, & Nanetti, 1993). Putnam's work is widely used in health research and population studies.

Given its multidisciplinary nature and the lack of consensus on its definition, an ongoing debate has led to the development of two different but overlapping ways of approaching SC in the health field (Moore & Kawachi, 2017). The “cohesion” approach defines SC in terms of the resources available to members of social groups (Kawachi, 2006; Moore, Shiell, Hawe, & Haines, 2005; Portes, 1998). In contrast, the “network” approach emphasizes the fact that those resources are embedded within an individual's social network (Kawachi, 2006; Lin, 1999). As suggested by other authors, these two concepts are not contradictory, nor do they detract from the importance of recognizing that social resources can have an impact on individual and, ultimately, on public health (Kawachi, 2006; Moore & Kawachi, 2017).

## 3 THE MANY FORMS OF SOCIAL CAPITAL

**Figure 1:**
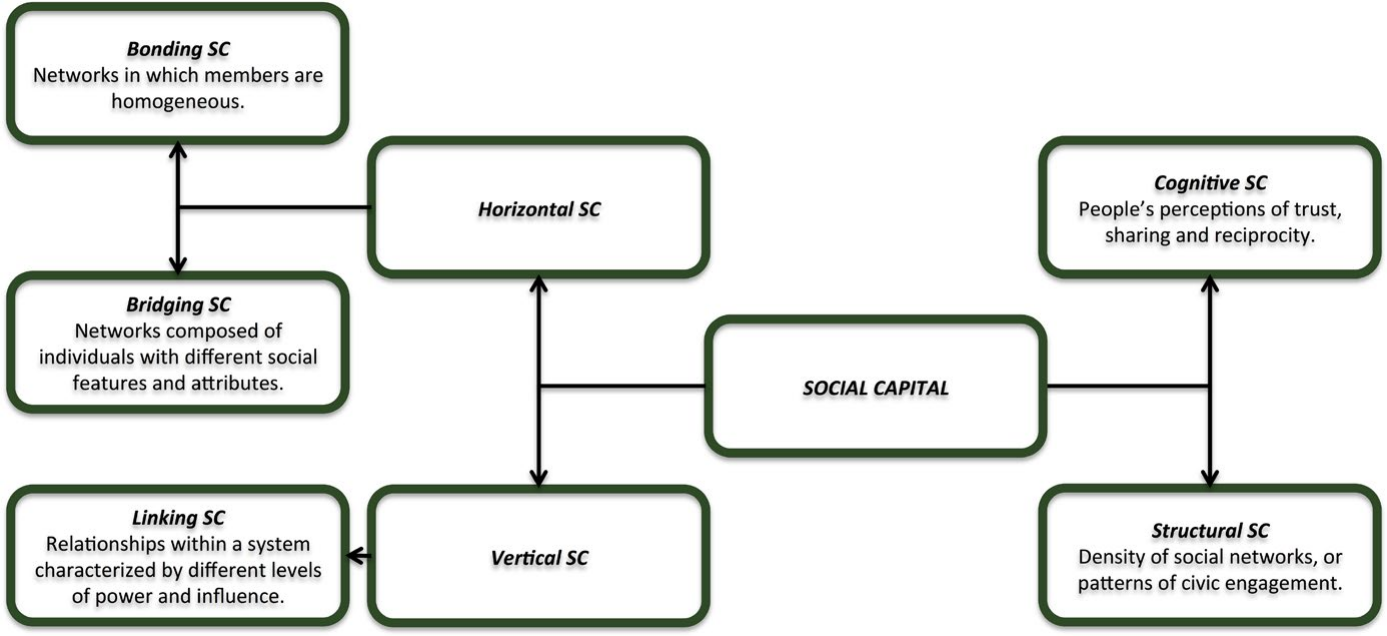
Forms and dimensions of SC. This figure was reproduced and modified from Islam et al. (2006). SC: social capital

Many types of SC are theoretically possible. Accordingly, there are different forms of operationalization of the concept (Figure 1). In order to embrace the entire phenomenon in all its complexity, it is necessary to look more closely at its major dimensions.

The first distinction that should be made is between the structural and cognitive component of SC. The structural dimension is derived from the “visible” forms of SC and consists of networks, relationships, associations, institutions, and organizations that link individuals and communities. On the other hand, the cognitive component refers to the quality of those social structures in terms of people's perceptions of trust, sharing, and reciprocity (Harpham, Grant, & Thomas, 2002; Krishna & Uphoff, 1999; McKenzie, Whitley, & Weich, 2002). Most studies in the field have used both forms of SC, based on the differential relationship of each type with health outcomes (Harpham, 2008).

Based on the direction of social resource flow and on the hierarchical relationships among individuals, a second classification model differentiates between bonding, bridging, and linking SC.

Bonding SC refers to the internally oriented networks in which members are homogeneous, and perhaps reinforces exclusive social identities in relation to other group identities and outsiders alike. The “bonding” dimension, as proposed by Putnam, may strengthen specific reciprocity and bolsters solidarity (Gittell & Videl, 1998; Putnam, 2000). In contrast, bridging SC refers to the more “diverse” outward‐oriented networks, composed of individuals with different social features and attributes. Because of its nature, the “bridging” dimension would facilitate access to external resources and the sharing of information (Gittell & Videl, 1998; Putnam, 2000). More recently, another form of SC has emerged. The “linking” dimension refers to norms of respect and trusting relationships within a status system, with players characterized by different levels of power and influence (Szreter & Woolcock, 2004). Seen from a different perspective, bonding and bridging SC are derived from horizontal relations of cooperation, while the linking SC is related to the vertical relations of authority (Gittell & Videl, 1998; Szreter & Woolcock, 2004). Some authors argue that linking SC is a subset of bridging SC. Although the three dimensions share many similar traits, it is important to address each component thoroughly to better elucidate their positive or negative consequences on people's well‐being (Kawachi, 2006).

Social capital can be further broken down and operationalized into three main levels, a “macro” level, where historical, political, and economic factors interact and influence the production and distribution of SC; a “meso” level where neighborhoods’ characteristics modify the dynamics of social relationships and consequently affect the traffic of SC within communities; and finally, a “micro” level that includes individual‐level variables and can be divided into two types: one that focuses on the individual behavior of the members within a community, and a second that emphasizes the psychological constructs of SC in terms of humans’ attitudes and beliefs, including trust in neighbors, trust in government, and expectations of reciprocity (Macinko & Starfield, 2001). At the risk of oversimplifying, there are external or “ecological” forces that engender SC processes, as well as internal or “individual” factors (Portes, 1998; Putnam et al., 1993). The different levels of SC are not necessarily mutually exclusive; they are all immersed within a multilevel analytical framework (Giordano, Ohlsson, & Lindström, 2011; Kawachi, Kim, Coutts, & Subramanian, 2004; Whitley & McKenzie, 2005).

## 4 MEASUREMENT OF SOCIAL CAPITAL

Despite the wide use of the concept of SC in public health over the last two decades, there is no standardized method to assess the impact of SC on health‐related outcomes (Moore & Kawachi, 2017). While efforts have led to conceptualizations and different classification systems that provide some relief to the process, they also, perhaps paradoxically, have increased the challenge to develop tools that fully address the multidimensional nature of SC. As many social aspects are encompassed under the same concept, over‐simplification and over‐standardization are both equally dangerous for SC research.

As previously mentioned, two major approaches to SC have been described. From a “cohesion” perspective, researchers have sought to measure SC through surveys inquiring about trust in others, perceptions of social belonging, shared norms and levels of civic participation and social interaction (Moore & Kawachi, 2017). Specific instruments such as the SC Assessment Tool (SCAT) and its adapted (ASCAT) and shortened (SASCAT) versions have been developed to aid this process. These tools have the unique advantage of clearly distinguishing between cognitive and structural components of SC, which allows analysis of the independent associations of these components with health variables (Harpham et al., 2002). However, their reliability has not been well established (Agampodi, Agampodi, Glozier, & Siribaddana, 2015).

Three main instruments have been employed to construct “network” measures of SC: name, position, and resource generators. The “name generator” method consists of asking participants to list all persons to whom they are related in certain social contexts or situations. From these data, network locations and social resources can be computed. Another instrument is the “position generator,” which consists of asking participants whether they personally know someone in a number of salient social positions, usually occupations. From the responses, it becomes possible to construct SC indexes such us extensity, heterogeneity, and reachability of an individual's social network (Lin, 2001; Lin, Cook, & Burt, 2001; Van Der Gaag & Webber, 2008). While the application of these instruments contributes to valuable research findings, they also have specific flaws. The lack of consistency in the way name generator data have been obtained and analyzed makes comparisons and validation impossible. On the other hand, the position generator data have been criticized for not providing specific information about the diversity of the accessed SC (Lin, 2001; Lin et al., 2001; Van Der Gaag & Webber, 2008). The “resource generator” method has been introduced in an effort to overcome the inherent limitations of earlier techniques. This instrument asks whether the participants personally know someone who can give them access to a fixed list of resources, and examines the tie role through which these specific resources are available. From these data, SC measures are constructed based on the assumption that SC resources are generally better available through stronger ties. Although the “resource generator” instrument can result in valid and easily interpretable representations of SC, it has yet to be fully integrated into health research (Moore & Kawachi, 2017; Van Der Gaag & Snijders, 2005).

A well‐known controversy in the research of SC surrounds the level at which it should be measured: as an individual or as a group attribute. Some authors have highlighted that the novel contribution of SC lies in its collective level, arguing that this approach will better differentiate the SC theory from the previously established and conceptually different association between social networks/support and health‐related outcomes (Giordano et al., 2011; Whitley & McKenzie, 2005). As might be expected, an intuitive approach has been commonly used in the analysis of collective SC, by aggregating the individual SC scores collected from a representative sample of the community. Other methods such as the per capita number of public places and the voting rates have also been used (Ehsan & De Silva, 2015). The challenge would be to develop a valid and reliable tool for the evaluation of collective SC, as aggregated individual measures may not genuinely reflect SC at higher contextual levels (Poortinga, 2006). Whether it is measured as an individual characteristic or as a party attribute, this should be justified in advance and oriented to develop measures at these various levels.

## 5 SOCIAL CAPITAL AND HEALTH

The relocation of SC from the social theory to the public health arena has been attributed, at least in part, to two factors: (a) in response to earlier observations that social networks are powerful determinants of health outcomes; and (b) as one potential mechanism through which income inequality affects population health and mortality (Pearce & Davey Smith, 2003).

A vast literature has linked social networks to health (Smith & Christakis, 2008). For example, extensive data from cross‐sectional studies have shown that married persons have significantly lower mortality than the unmarried. Indeed, a short‐term rise in mortality following the loss of a spouse has also been demonstrated (Elwert & Christakis, 2008; Lillard & Panis, 1996). Additionally, there is evidence of numerous nonspousal interpersonal health effects. For instance, maternal depression has been associated with negative psychosocial outcomes in children, including behavioral problems, depression, and substance abuse. In another example, the smoking behavior of friends is a major risk factor for adolescent smoking uptake (Chen, White, & Pandina, 2001; Colletti et al., 2009; Smith & Christakis, 2008). Although important, social networks are just one component of the SC realm.

In an effort to test whether SC explained the associations between income inequality and health, Kawachi et al. (1997) performed a cross‐sectional study in the United States based on data from 39 states. SC was measured by the per capita density of membership in voluntary groups in each state and the level of social trust. The income inequality was strongly correlated with SC indicators. Similarly, SC measures were associated with mortality rates. Their ecological approach suggested that income inequality exerts a large indirect effect on overall mortality through the SC variable.

In another classic study, Sampson et al. explored an additional role for SC in the determination of community well‐being (Sampson, Raudenbush, & Earls, 1997). The study assessed whether “collective efficacy” mediated the association of residential instability and disadvantage with rates of interpersonal violence at the neighborhood level. Collective efficacy was determined by using the proxies of “social cohesion” and “informal social control,” which were measured by 5‐point Likert scales. Their analysis showed that collective efficacy was a robust predictor of lower rates of violence (Sampson et al., 1997).

In addition to these reports, many researchers have theoretically addressed as well as empirically assessed the links between SC and health. Their findings traced the gradual accrual of evidence supporting the protective effect of SC on mental and physical health and mortality (Almedom, 2005; Holt‐Lunstad, Smith, & Layton, 2010; Kim, Subramanian, & Kawachi, 2008; Schultz, O'Brien, & Tadesse, 2008). Nevertheless, the strength of associations varies between constructs and again between levels of measurement.

Accumulating evidence has shed new light on the theoretical model linking SC to health. Kawachi and Berkman (2014) have proposed a plausible framework that encompasses individual and group‐level pathways while incorporating the “public good” aspect of SC. At the individual level, the relevant mechanisms include the acquisition of useful health‐related information, the gaining of instrumental support and social reinforcement. The processes that underlie this relationship at the group level involve the diffusion of appropriate behaviors through tightly knit social networks, the ability of the community to suppress deviant behaviors, and the collective efficacy to undertake collective actions. The “public good” theory refers to the parallel benefits of SC for people beyond the boundaries of immediate networks of interaction. In other words, individuals cannot be prevented from accessing these social goods (Kawachi & Berkman, 2014). This approach perfectly fits the concept of SC as somewhat intangible and context‐dependent. It is hence interesting to explore whether SC as a public good is not only non‐excludable but also nonrival.

Social capital does not exist in a vacuum; it is embedded in structural contexts. It is crucial to recognize that the effects of SC on health are also shaped by economic, political, and other material factors (Lynch, Smith, Kaplan, & House, 2000; Pearce & Davey Smith, 2003; Pilkington, 2002).

## 6 SOCIAL CAPITAL INTERVENTIONS

Existing and emerging evidence on SC and health needs to be used and translated into interventions. Given that this is still an immature field, there is no specific recipe for advancing SC interventions and then implementing, sustaining, and evaluating them in real‐world settings. As a first step, Moore, Salsberg, and Leroux (2013) have proposed a set of methodological principles for developing SC interventions. While this theoretical approach highlights that researchers should address the sources of SC as part of the intervention, it also suggests that those interventions should aim to reduce SC inequities.

Few publications have focused on SC interventions and health outcomes. Although the evidence derived from these observational studies yielded mixed results, a more recent work by Coll‐Planas et al. (2017) highlighted the potential of SC interventions to reach comprehensive health effects. The authors conducted a systematic review of controlled trials to assess the health impact of SC interventions targeting older people. The included studies were very heterogeneous preventing a meta‐analysis, and the risk of bias was high or unclear for all but eight trials. However, when focused on studies judged as high quality, SC interventions showed a favorable effect on overall, mental and physical health, mortality, and use of health‐related resources (Coll‐Planas et al., 2017). This raises the question of whether there is no compelling evidence for the impact of SC interventions on health outcomes, or whether it has not been properly studied yet. Despite the need for higher quality research, this review can be used as an evidence base to support SC interventions from a public health perspective, including people with neurological disease (Coll‐Planas et al., 2017).

## 7 NEGATIVE SOCIAL CAPITAL

The potential negative effects of SC were recognized in the sociological literature before the concept became popular in public health research; however, they are often superficially documented or not explored in detail (Portes, 1998; Portes & Landolt, 1996). Portes (1998, 2014) have provided some balance to the discussion by reviewing such less desirable consequences that include the following: (a) The ties that bring benefits to members of a group may ban outsiders from the same resources; (b) excess claims on successful group members; (c) social participation necessarily creates demands for conformity and improperly constrains individual freedom; and (d) downward leveling norms that may perpetuate a group's subordinate status. It has also been proposed that reciprocity norms elicit and implicit obligation to return actions or services provided by another person. Although such exchanges may be beneficial to individuals with respect to health outcomes, they may also exhibit negligible or detrimental effects, provoking feelings of inadequacy or dependence (Perry & Pescosolido, 2015).

Despite the scarce evidence, the potential downside of SC should be recognized as a phenomenon worthy of study. If not properly addressed, SC interventions could fail to move from theory to practice in ways that truly promote health (Moore, Daniel, Gauvin, & Dubé, 2009; Villalonga‐Olives & Kawachi, 2017; Wakefield & Poland, 2005).

## 8 SOCIAL CAPITAL AND NEUROLOGICAL CONDITIONS

Social networks and interpersonal interaction are critically involved in neurobiological processes (Dhand et al., 2016). This has been suggested in the literature both from animal models, showing that social isolation affects neuroplasticity in the mature brain, and from observational studies, showing that loneliness is associated with an increased risk of late‐onset dementia and correlates with worse outcomes after stroke (Cacioppo, Fowler, & Christakis, 2009; Ieraci, Mallei, & Popoli, 2016; Wilson et al., 2007).

There is a growing body of evidence that social stress leads to inflammation, oxidative stress, and neurodegeneration (Schiavone et al., 2009). Several studies have demonstrated that social isolation induces neuroinflammatory changes and the associated cellular response of microglia. The ionized calcium binding adaptor molecule 1 (Iba‐1), a microglial marker, has been found to be elevated in the hippocampus and prefrontal cortex of isolated rats. Interestingly, the expression of glucocorticoid receptors in the brain is greatest within those regions, making them particularly sensitive to stress‐inducing stimuli (Calcia et al., 2016). In a similar manner, the superoxide‐producing nicotinamide adenosine dinucleotide phosphate oxidase 2 is highly expressed in rats exposed to social isolation, thereby causing an increase in oxidative stress in the central nervous system (Schiavone et al., 2009). Loneliness has even been associated with changes in brain plasticity. Reduction in several neuroplasticity‐related genes has been documented in the brain of socially deprived mice. Specifically, a down‐regulating effect on a variant of the neuroplasticity marker brain‐derived neurotrophic factor has been observed (Ieraci et al., 2016). Isolation has also been shown to delay the positive effect of physical activity on adult neurogenesis. Accordingly, it has been hypothesized that social experience prevents endogenous glucocorticoids from suppressing neuronal proliferation (Stranahan, Khalil, & Gould, 2006).

Within the clinical literature and as previously reported for other cardiovascular conditions, a recent study exploring the longitudinal association between social support and risk of stroke found that having a small social network was associated with a modestly increased risk of incident stroke (Nagayoshi et al., 2014). Additional evidence also suggests that prestroke social isolation may contribute to poorer stroke outcomes including stroke recurrence, myocardial infarction, and death, due to poor compliance, depression, and stress (Boden‐Albala, Litwak, Elkind, Rundek, & Sacco, 2005). A similar effect has been postulated to modify the risk of dementia. Authors of a systematic review on the association between lifestyles and cognition concluded that a socially integrated lifestyle in late life seems to protect against Alzheimer's disease (AD) (Fratiglioni, Paillard‐Borg, & Winblad, 2004). Another study showed that the extent of social networks modified the relation between the density of neurofibrillary tangles and cognitive function assessed proximate to death, suggesting that social support provides some type of reserve which reduces the deleterious effect of AD pathology on cognitive abilities (Bennett, Schneider, Tang, Arnold, & Wilson, 2006).

Bringing patients’ social context into medical interviewing is one of the foundations of medical care and seems to be underpinned by an emerging biological evidence base. Dhand and colleagues have recently proposed a comprehensive framework for advancing social network science in neurology, which involves the characterization of social networks in patients with common neurological disorders, the identification of the mechanisms regulating the interplay between the social networks and neurological outcomes, and finally the evaluation of potential network interventions (Dhand et al., 2016). This approach could be easily generalized to other specialties and subspecialties. As previously mentioned, social networks are important but just one piece of the SC puzzle.

There is no doubt that social factors affect patients with neurological conditions. However, only very few empirical studies have directly investigated the relationship between SC and neurological outcomes.

In terms of cognitive health, Murayama et al. (2013) examined the association of bonding and bridging SC with cognitive decline and other health outcomes in older Japanese. Five‐point Likert scales at the individual level were used to measure both dimensions of SC: bonding SC in terms of perceived neighborhood/network homogeneity and bridging SC as perceived network heterogeneity. The results showed that neither bonding nor bridging SC was associated with cognitive decline. Several strengths of this research are evident, including its longitudinal nature and the use of SC measures that correspond to the theoretical framework previously described in the literature. However, other core aspects of SC such as the levels of participation in organizations were not evaluated and might have affected their findings and conclusions. In contrast, another study conducted in China found an inverse association between bonding SC and mild cognitive impairment. The authors hypothesized that bonding SC is more important for Chinese elderly people because they mainly receive care from their families and not from their bridging networks (Wang et al., 2016). However, its cross‐sectional design prevents the direction of the association from being determined. More recently, Hikichi et al. (2017) took advantage of a unique natural experiment to capture the buffer effect of SC on threats to cognitive function. This research prospectively examined the association between changes in SC and cognitive function, before and after the 2011 great east Japan earthquake and tsunami. The authors focused on the cognitive and structural components of individual SC. The results showed that experiences of disaster are associated with an increased risk of cognitive decline, while SC seemed to mitigate that effect. These findings have crucial implications for community action in the aftermath of disasters. Finally, there is also evidence indicating that SC is associated with cognitive development and recovery trajectories after pediatric traumatic brain injury (Caughy & O'Campo, 2006; Keenan, Clark, Holubkov, Cox, & Ewing‐Cobbs, 2018; Keenan, Hooper, Wetherington, Nocera, & Runyan, 2007; Keenan, Runyan, & Nocera, 2006; Runyan et al., 1998).

Some studies have focused on the influence of SC on quality of life. In a recent cross‐sectional survey conducted in Iran, researchers explored the association between SC and quality of life among patients with multiple sclerosis (MS). In contrast to previous studies, the authors used a more comprehensive tool to measure several dimensions of SC. The analysis showed that SC and quality of life are certainly dependent and might have a positive effect on each other (Rimaz, Mohammad, Dastoorpoor, Jamshidi, & Majdzadeh, 2014). However, it is worth mentioning that they did not formally control for other potential confounding factors such as depression, which has been shown to be a major determinant of quality of life in MS patients (Amato et al., 2001; Lobentanz et al., 2004). Last but not least, SC has also been shown to play a role in sleep quality (Bassett & Moore, 2014; Nieminen et al., 2013; Nomura, Yamaoka, Nakao, & Yano, 2010; Takahashi et al., 2014).

Despite the balance of current evidence tilts toward a beneficial effect of SC on patients with neurological disorders, no clear picture emerges and more research is warranted. We hypothesized that SC may have both positive and negative effects on the outcomes of these patients. This hypothesis draws attention to the theoretical assumption that SC interventions are also associated with both benefits and drawbacks to different groups. As stated before, the challenge would be to translate the emerging evidence into interventions for health‐promoting purposes. The question remains open and its relevance is just beginning to be acknowledged. An interesting initiative was the development of the Keep Moving toward Healthy Heart and Healthy Brain (KM2H^2^) for the prevention of heart attack and stroke. The KM2H^2^ is a behavioral intervention that, guided by the SC theory, motivates physical activity by increasing access to the social and emotional resources. A recent study shed a light on its effectiveness for use in community settings (Gong, Chen, & Li, 2015).

## 9 IMPLICATIONS FOR THE NEUROLOGIST

**Figure 2:**
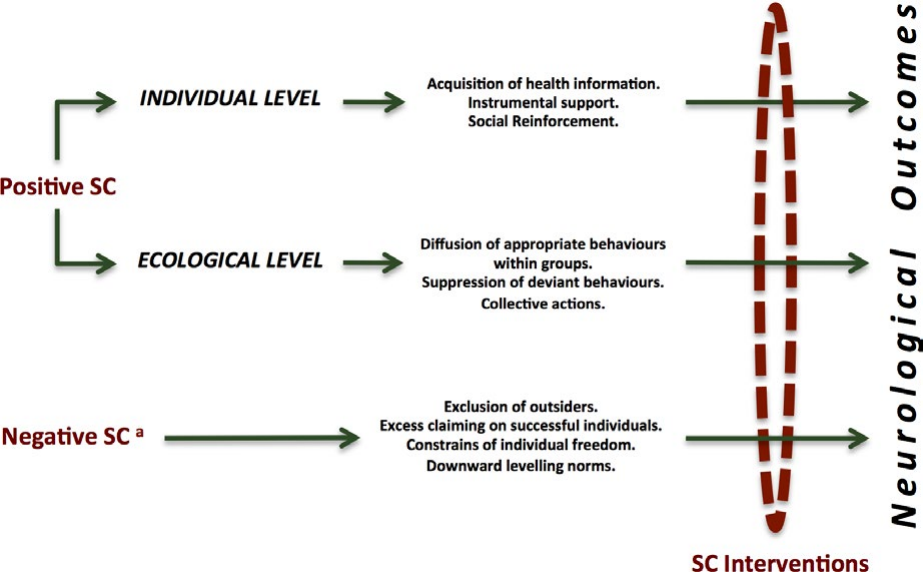
Social capital (SC) environment of a neurological patient and its links to health outcomes. This figure is based on the theoretical framework reviewed earlier (Islam et al., 2006; Kawachi & Berkman, 2014; Portes, 2014; Villalonga‐Olives & Kawachi, 2017). ^a^Further work is needed to analyze the potential downside of SC from an individual and ecological level. Similar mechanisms could have both positive and negative effects on individual and community health

As understanding of SC extends beyond the mere relationship between social support/networks and health‐related outcomes, the theory must expand to encompass the role of SC in neurology. Rather than offering an exhaustive guideline to approach SC in patients with neurological conditions, in Figure 2 we attempt to represent how our patients are embedded in their SC environment. The suggested framework is based on both the social cohesion and the network definitions of SC. From an empirical perspective, and considering the aforementioned evidence that SC interventions might promote general health (Coll‐Planas et al., 2017), we proposed that SC constitutes an integral part of medical care for neurological patients. As SC is already happening, a reasonable starting point would be to observe how the patients make use of their SC resources and interact with the different SC actors. As a result, neurologists would learn about the dynamics of SC simply from observation during their daily clinical practice. This model is not intended to be prescriptive, but to help practitioners more effectively monitor their patients’ SC background and act accordingly.

Social capital interventions should involve either a change in social structures or a behavioral induction, as Villalonga‐Olives, Wind, and Kawachi (2018) have previously suggested. Some strategies that have been shown to increase SC in the domain of public health may also be used in patients with neurological conditions to influence health outcomes. These interventions may include encouraging patients to volunteer in their local community, engage in group‐based physical activity programs, and participate in social groups focusing on particular interests, such as reading (Villalonga‐Olives et al., 2018).

Further research using longitudinal data and multilevel approaches is needed to continue refining SC measurement in neurological patients. Accordingly, SC should be included as a covariate in future studies of quality of life and health‐related outcomes. This would also help in the development of more precise SC interventions as has occurred in other areas of medicine.

## 10 CONCLUSION

Social capital is a multidimensional phenomenon that health researchers have adopted from the sociology theory. Despite the controversy concerning its conceptualization and operationalization, accumulating evidence has shed new light on the links between SC and health. As the SC theory develops, many debates will continue, and more questions are likely to be raised. It is foreseeable, particularly in view of the fact that SC attempts to cover such a variety of social phenomena. Its precise role in neurology is just beginning to be elucidated, and more research is needed to move forward by integrating theory and empirical evidence into clinical practice.

## CONFLICT OF INTEREST

SR declares no conflict of interest. GG has been a consultant and served on scientific advisory boards for AbbVie, Atara Biotherapeutics, Biogen, Canbex, Five Prime, Genentech, Genzyme, GSK, GW Pharma, Ironwood, Merck‐Serono, Novartis, Roche, Sanofi Genzyme, Synthon BV, Teva, and Vertex Pharmaceuticals. GG serves as chief editor for Multiple Sclerosis and Related Disorders. AT has received speaker honoraria from Novartis and has received unrestricted educational grants from Biogen, Genzyme Sanofi, Novartis, and Roche.
